# Gene amplification of the *Hps *locus in *Glycine max*

**DOI:** 10.1186/1471-2229-6-6

**Published:** 2006-03-14

**Authors:** Mark Gijzen, Kuflom Kuflu, Pat Moy

**Affiliations:** 1 Agriculture and Agri-Food Canada, 1391 Sandford Street, London, Ontario, N5V 4T3, Canada

## Abstract

**Background:**

Hydrophobic protein from soybean (HPS) is an 8 kD cysteine-rich polypeptide that causes asthma in persons allergic to soybean dust. HPS is synthesized in the pod endocarp and deposited on the seed surface during development. Past evidence suggests that the protein may mediate the adherence or dehiscence of endocarp tissues during maturation and affect the lustre, or glossiness of the seed surface.

**Results:**

A comparison of soybean germplasm by genomic DNA blot hybridization shows that the copy number and structure of the *Hps *locus is polymorphic among soybean cultivars and related species. Changes in *Hps *gene copy number were also detected by comparative genomic DNA hybridization using cDNA microarrays. The *Hps *copy number polymorphisms co-segregated with seed lustre phenotype and HPS surface protein in a cross between dull- and shiny-seeded soybeans. In soybean cultivar Harosoy 63, a minimum of 27 ± 5 copies of the *Hps *gene were estimated to be present in each haploid genome. The isolation and analysis of genomic clones indicates that the core *Hps *locus is comprised of a tandem array of reiterated units, with each 8.6 kb unit containing a single HPS open reading frame.

**Conclusion:**

This study shows that polymorphisms at the *Hps *locus arise from changes in the gene copy number via gene amplification. We present a model whereby *Hps *copy number modulates protein expression levels and seed lustre, and we suggest that gene amplification may result from selection pressures imposed on crop plants.

## Background

The lustre or glossiness of soybean seeds is a variable trait that is controlled by genetic and environmental factors [1,2]. The amount of endocarp adhering to the seed surface is the primary determinant of lustre [3,4]. The presence of adhering endocarp tissues also lightens the colour of the seed and produces soybeans with a paler or more whitish appearance. This is equally true for pigmented soybeans as for yellow or buff coloured soybeans that lack seed coat pigmentation. A dense or contiguous covering of the honeycomb-like endocarp tissue produces a bloom phenotype, whereas a fragmented or patchy covering of endocarp produces a dull phenotype [5]. Shiny phenotypes occur when seeds are mostly free of endocarp deposits on the surface. In a cross between dull- and shiny-seeded phenotypes, dull-seededness segregates as a single dominant gene *B *[2]. Additional genes that influence seed lustre have also been proposed [1,6,7].

It is not known what molecules control the adherence of endocarp to the seed surface, but one likely factor is an 8 kDa cysteine-rich protein named HPS (hydrophobic protein from soybean). Past studies indicate that HPS is synthesized in the endocarp and deposited on the seed surface during development [5]. The presence of HPS on the seed surface is a trait that cosegregates with the seed lustre determinant *B *[2]. These facts along with other evidence suggest that HPS can mediate the attachment of endocarp tissues to the seed surface and thereby affect the seed lustre.

The HPS protein has also been named Gly m 1 because it is the major allergen that causes asthma in persons allergic to soybean dust [8]. Epidemic outbreaks of asthma caused by the presence of soybean dust have been documented in many cities [9]. The occurrence of relatively large amounts of HPS on the seed surface results in the release of aerosols containing the protein during seed handling. Airborne HPS can be detected in ports where soybeans are transferred and even in regions where soybeans are grown, during the harvesting season [10,11].

Here we demonstrate that genetic polymorphisms that affect the copy number of the *Hps *gene are prevalent in soybean germplasm. We show that *Hps *genes are clustered in a tandem array at a single genetic locus, and we suggest that a process of gene amplification has led to this structural arrangement. Finally, we propose that changes in *Hps *gene copy number modulate HPS protein expression levels and seed lustre phenotypes.

## Results

### The *Hps *gene structure is polymorphic among soybean cultivars

**Figure 1:**
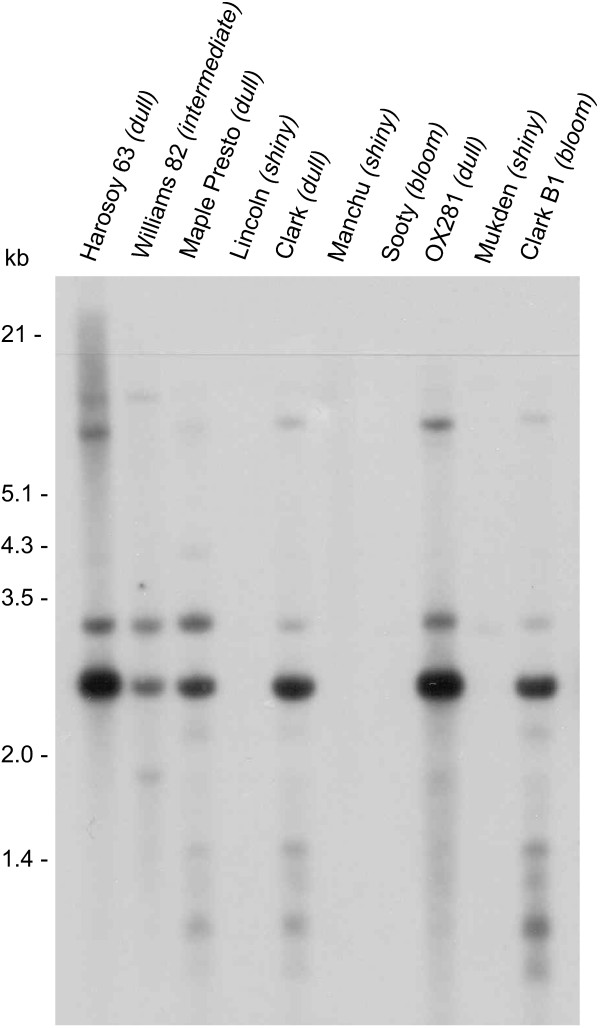
**Analysis of *Hps *gene structure in various soybean cultivars and lines by DNA blot hybridization. **Soybean (*Glycine max*) genomic DNA (30 μg each lane) was digested with *Bgl *II and separated by agarose gel electrophoresis. The DNA was transferred to Nylon blotting membrane and probed with ^32^P labelled *Hps *cDNA (GenBank accession number AF100159). The name of the soybean cultivar or line is shown above each lane along with the seed lustre phenotype in parenthesis. The migration of DNA size standards is shown on the left.

To compare *Hps *gene structure among soybean cultivars or lines that differ in seed lustre, a DNA blot analysis was performed using an *Hps *cDNA probe. Figure 1 shows results from a representative analysis of ten different soybean lines, after digestion of genomic DNA with the restriction enzyme *Bgl *II. Polymorphisms were noted in both the number and intensity of hybridizing genomic DNA fragments among the different cultivars and lines. The most intensely hybridizing fragment was estimated to be 2.4 kb in size. This fragment could produce strong hybridization signals even after short exposure times, indicating that multiple copies may be present in genomes of selected soybean cultivars or lines. The presence of this hybridizing fragment was associated with seed phenotypes that were dull or intermediate in lustre. This fragment was absent from shiny seeded phenotypes. Two different soybean lines with a bloom phenotype showed contrasting patterns, with the hybridizing band present in Clark *B1 *but absent from Sooty.

**Figure 2:**
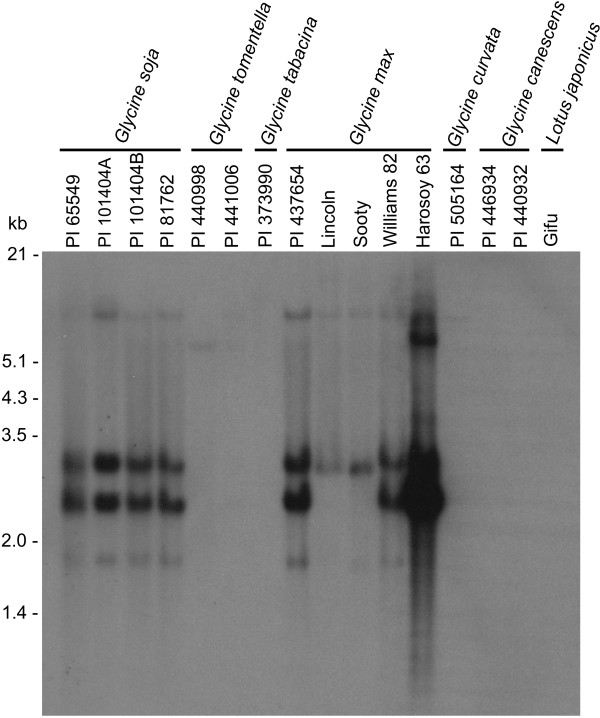
**Hybridization of *Hps *cDNA to genomic DNA from seven different legume species. **Genomic DNA (30 μg each lane) was digested with *Bgl *II and separated by agarose gel electrophoresis. The DNA was transferred to Nylon blotting membrane and probed with ^32^P labelled *Hps *cDNA (GenBank accession number AF100159). The name of the line or cultivar is shown above each lane along with species name in italics. The migration of DNA size standards is shown on the left.

This analysis was extended to compare soybean DNA samples to genomic DNA samples isolated from six related legume species, including five species from the same genus *Glycine*. The results shown in Figure 2 indicate that hybridizing bands to *Hps *cDNA could only be detected in two species, soybean (*Glycine max*) and wild soybean (*Glycine soja*), at least under the high-stringency hybridization conditions that were performed here. The genomic DNA samples from the five different soybean lines displayed greater differences in their hybridization patterns and signal intensities than did the four *Glycine soja *lines. The strong hybridization signals present in *Glycine max *cv Harosoy 63 were not detected in any of the *Glycine soja *lines, although the patterns of hybridization were similar. The hybridization results shown in Figure 2 also demonstrate that copies of the *Hps *gene are present in shiny seeded phenotypes.

### Multiple copies of *Hps *are present at a single genetic locus

**Figure 3:**
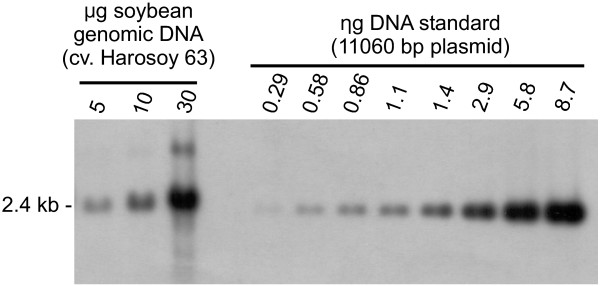
***Hps *gene copy number determination by reconstruction hybridization. **Soybean (*Glycine max *cv. Harosoy 63) genomic DNA, or an 11060 bp plasmid carrying a soybean chromosome fragment encoding a single copy of *Hps *(GenBank accession DQ208939) were digested with *Bgl *II and separated by agarose gel electrophoresis. The DNA was transferred to Nylon blotting membrane and probed with ^32^P labelled *Hps *cDNA (GenBank accession number AF100159). Shown above each lane is the amount of DNA loaded for the soybean genomic DNA (μg) or for the plasmid standard (ng). The size of the main hybridizing fragment is indicated on the left. Analysis of the band intensities using a phosphoimager results in a calculated value of 27 ± 5 copies of *Hps *per haploid genome, for the 2.4 kb *Bgl *II fragment.

The restriction enzyme fragments producing strong hybridization signals in the DNA blot analyses suggested that *Hps *may occur as a multi-copy gene in certain *Glycine max *lines, such as cv Harosoy 63. To estimate the number of copies of the *Hps *gene present in soybean cv Harosoy 63, hybridization signals were compared between samples of soybean genomic DNA and a plasmid standard carrying a single copy of the *Hps *gene. Results from this 'reconstruction hybridization' are shown in Figure 3. Measurement of the band intensities by image analysis results in a calculated value of 27 ± 5 copies of *Hps *per haploid genome, for the 2.4 kb *Bgl *II fragment, assuming a haploid genome size of 1.212 pg DNA [12]. This is a minimum value, since additional bands hybridizing to the *Hps *cDNA were also present in the *Bgl *II digestions of cv. Harosoy 63.

**Figure 4:**
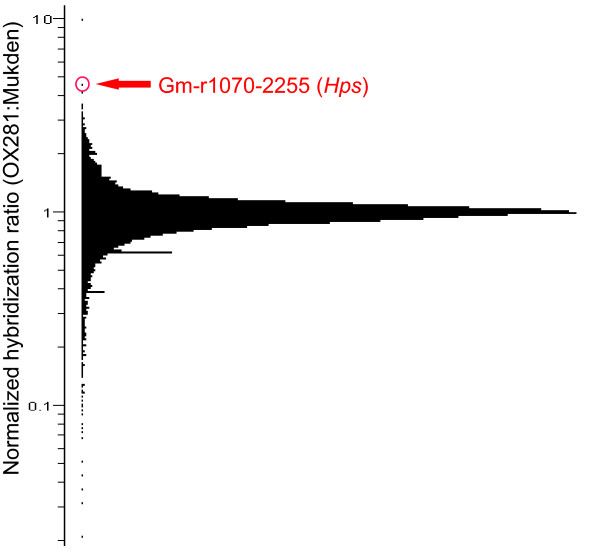
**Microarray analysis of gene hybridization ratios comparing genomic DNA from soybean lines polymorphic for *Hps*. **Genomic DNA samples from soybean cv Mukden and from line OX281 were individually labelled with fluorescent dyes Cy3 and Cy5, mixed, and hybridized to an 18 K array of soybean cDNAs. Genes from the array are plotted according to their normalized hybridization ratios (GeneSpring 7.2; Silicon Genetics, Redwood City, CA). The spot on the array corresponding to cDNA clone Gm-r1070-2255 encodes *Hps*. Data from a representative experiment is shown (Experiment II), where normalized hybridization values are averaged from separate hybridizations after swapping fluorescent dye labels for each genomic DNA sample.

To determine whether differences in *Hps *gene copy number among soybean lines could be detected by other methods, we performed a comparative genomic DNA hybridization to cDNA microarrays. This method has been shown to be effective to distinguish changes in gene copy number in other species [13]. A total of six hybridizations were performed in three experiments, using a cDNA microarray of 18613 soybean cDNAs. Two experiments compared genomic DNA from OX281 to Mukden, and one experiment compared genomic DNA from Harosoy 63 to Sooty, as shown in Table 1. The results from Experiment II are shown in more detail in Figure 4.

The absolute hybridization values, and therefore the signal-to-noise ratios, were low compared to microarrays that were conventionally probed with cDNA derived from mRNA samples. Nonetheless, the normalized ratios for the ~18,000 genes on the array were tightly clustered around unity (~1), as expected, but the cDNA on the array encoding HPS displayed exceptional hybridization ratios in these experiments in every case. Thus, in each experiment that compared hybridization ratios of OX281:Mukden or of Harosoy 63:Sooty, the cDNA encoding HPS always displayed a hybridization ratio >2, ranging from 4.2 to 6.4. These results indicate that the *Hps *copy number differences detected by conventional Southern analysis are also detectable by comparative genomic DNA hybridization to cDNA microarrays.

**Figure 5:**
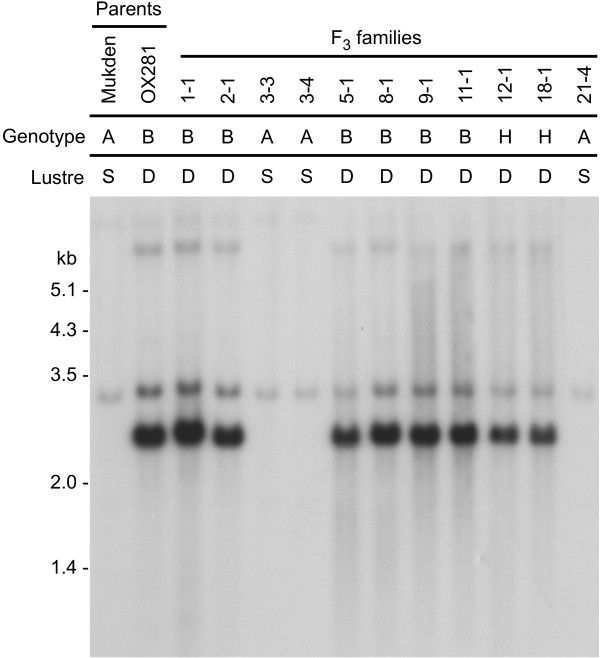
**Segregation of *Hps *polymorphisms and seed lustre in a soybean cross. **An analysis of a genetic cross between two different soybean lines polymorphic for *Hps *and seed lustre phenotype. Soybean (*Glycine max*) genomic DNA (30 μg each lane) was digested with *Bgl *II and separated by agarose gel electrophoresis. The DNA was transferred to Nylon blotting membrane and probed with ^32^P labelled *Hps *cDNA (GenBank accession number AF100159). Shown above each lane is: Lustre, the seed lustre for each parent or F_3 _family (F_4 _seed); Genotype, the genotype for molecular marker A963_CAPS [2] where A is homozygous for the Mukden allele, B is homozygous for the OX281 allele, and H is heterozygous. The parental names and F_3 _family numbers are also shown.

To determine whether the *Hps *copy number polymorphisms cosegregate with seed lustre phenotype, seed surface protein (HPS), and associated genetic markers [2], we analyzed 30 F_3 _families from a cross of OX281 and Mukden. A total of 8 of the F_3 _families were shiny in phenotype, without surface HPS, and displayed a non-repetitive, low-copy *Hps *restriction fragment length polymorphism (RFLP) pattern. The remaining 22 F_3 _families were dull in phenotype, with abundant surface HPS, and displayed a repetitive, high-copy *Hps *RFLP pattern. A representative hybridization is shown in Figure 5. As expected, results from this analysis indicate that *Hps *copy number polymorphisms absolutely cosegregate with seed lustre phenotype. This analysis additionally shows that the multiple copies of *Hps *that are present in OX281 segregate as a genetic unit, indicating that the copies occur together at a single locus.

### Analysis of genomic clones indicate a tandem array of *Hps *genes

**Figure 6:**
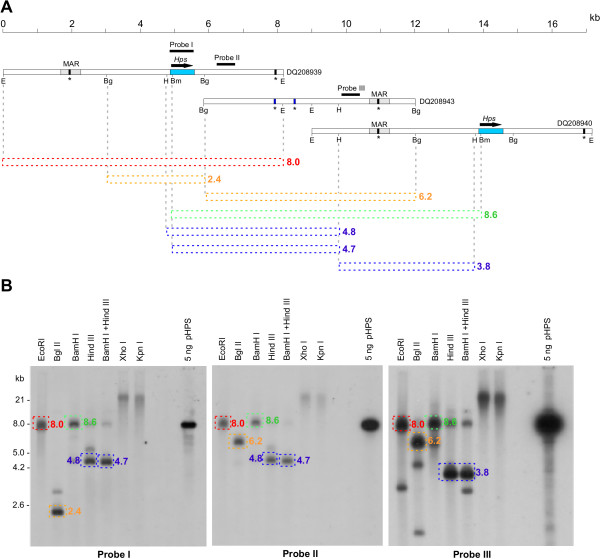
**An analysis of *Hps *genomic clones. **A, Three independent clones isolated from soybean (*Glycine max *cv. Harosoy 63) genomic libraries are illustrated, along with their GenBank accession numbers. Shown for each clone are: blue box, location of the *Hps *gene; arrow, direction of transcription; Bm, Bg, E, and H, restriction enzyme sites for *Bam*HI, *Bgl*II, *Eco*RI, and *Hin*dIII, respectively; MAR, predicted matrix attachment regions; * (asterik), location of micro-satellite dinucleotide AT repeats; Probe I, Probe II, and Probe III, correspond to DNA sequence fragments used to probe genomic DNA blots shown in the lower part of the figure. The three clones are aligned with overlapping regions of nearly identical DNA sequence. The predicted restriction enzyme fragments and their corresponding size (kb) are shown in colour directly below the genomic clones. B, Soybean (*Glycine max *cv. Harosoy 63) genomic DNA blot hybridizations using three different DNA probes, Probe I, Probe II, and Probe III. Samples of 20 μg of soybean genomic DNA were digested with the indicated restriction enzymes. A 5 ng sample of a plasmid (pHPS) carrying a genomic clone encoding *Hps *(GenBank accession number DQ208939) was digested with *Eco*RI, and included as an internal hybridization standard. The DNA samples were separated by agarose gel electrophoresis and blotted to Nylon membrane, prior to hybridization to each of ^32^P labelled DNA probes (with stripping between hybridizations). Coloured boxes and sizes (kb) of the most intense hybridizing fragments are indicated, and were matched to the predicted restriction enzyme fragments from the alignment of the genomic clones, shown in the upper part of the figure. The migration of DNA markers and their size (kb) are shown on the left.

To isolate the *Hps *gene(s), soybean genomic libraries were screened with an *Hps *cDNA probe. Additional probes, derived from the sequences of the genomic clones identified from the first round of screening, were used to isolate overlapping or flanking clones. In all, more than 30 genomic clones were isolated. The size, the library source, and the restriction enzyme digestion patterns of the various clones were compared. Using these criteria, the genomic clones could be classified into six different types. A representative of each type was chosen for complete sequencing, as shown in Table 2. Analysis of the DNA sequences revealed all of the clones shared regions of high sequence identity, and that three of the clones could be aligned to produce a repetitive motif, as shown in Figure 6. This hypothetical or model *Hps *repetitive motif was tested by DNA blot hybridization using three distinct probes derived from different regions of the repetitive unit. The results show that for each probe the most intensely hybridizing DNA fragments, representing most of the copies of the repetitive unit, could be accounted for by reconstructing the restriction enzyme fragments from the aligned genomic clones. A single *Hps *gene is present in each unit. A putative matrix attachment region was predicted to occur 3 kb upstream from the *Hps *open reading frame but no other genes were detected. Thus, most copies of *Hps *occur in a reiterated array of 8.6 kb units.

Other copies of *Hps *are also present in the soybean genome, as evidenced by the additional hybridizing bands on the DNA blots and by the genomic clones that do not exactly match the repetitive pattern. There are closely related genes, such as *HPS2.1*, that may correspond to paralogue pair-mates that arose from whole genome duplication, since soybean is considered an ancient tetraploid. Additional *Hps *genomic copies may also represent flanking regions or sequence variations occurring within the tandem array. For example, clones *HPS1.5 *(DQ208939) and *HPS1.6 *(DQ208940) share 97.5% sequence identity over their 8 kb length, but *HPS1.5 *has a single *Hin*d III site whereas *HPS1.6 *possess two *Hin*d III sites. These two copies of *Hps *will produce different patterns of hybridization after *Hin*d III digestion. Although both patterns appear to be visible in the DNA blots, the smaller sized fragments produce much stronger signals, indicating that clone *HPS1.6 *with two *Hin*d III sites better represents the majority of copies of *Hps *within the genome.

## Discussion

Past studies have pointed to a role for HPS in the control of seed lustre in soybean cultivars [2,5]. Now, we have conducted an extensive study of *Hps *copy number polymorphisms in a range of soybean lines and related legume species. The structure of the *Hps *gene was investigated by isolating and characterizing clones from the genomic region. The results have led us to propose a model to account for variation of seed lustre controlled by *Hps*.

From the analysis of DNA blot hybridizations of various soybean cultivars, lines, and related species, we can conclude that *Hps *copy number polymorphisms are common in soybean. The *Hps *locus appears to have evolved and diversified in soybean (*Glycine max*) in comparison to its wild ancestor (*Glycine soja*). Hybridization patterns show that the *Hps *sequence itself is also specific to these two species, a result that is supported by searches of DNA and protein sequences in GenBank (not shown). HPS shows similarities to so-called bi-modular proteins containing plant lipid transfer protein (LTP) domains [5]. The plant LTPs constitute a large group of related proteins derived from the prolamin super-family. Our results show that HPS has diverged substantially from other LTPs and that there are no close counterparts in other species.

All *Glycine max *lines that were tested contained multiple copies of the *Hps *gene, but there were large differences in the number of copies of *Hps *depending on the cultivar examined. We observed a good correlation between the apparent *Hps *copy number, as judged by hybridization intensity on DNA blots, and seed lustre. This is especially true for dull- and shiny-seeded phenotypes and for intermediates between these types. This relationship was not apparent for bloom phenotypes, an exception that has been noted in past studies that correlated the occurrence of HPS protein to seed lustre [2,5]. Two bloom phenotypes analyzed, Clark B1 and Sooty, produced contrasting patterns of *Hps *hybridization. This can be accounted for by tracing the pedigree of Clark B1. The cv Clark is a dull phenotype with a high-copy *Hps *RFLP pattern, whereas Sooty is a bloom phenotype with a low-copy RFLP pattern. Clark B1 is an isoline derived from a cross between Clark and Sooty, with Clark as the recurrent parent. This indicates that the bloom phenotype (*B1*) is controlled by genes that are independent of *B *and *Hps*.

Multiple copies of *Hps *could be detected in a number of different soybean cultivars and lines by conventional DNA blot hybridizations. Multiple genomic copies of *Hps *were also detected by real-time PCR analysis (not shown). Copy number estimates from real-time PCR analysis were more variable and always exceeded estimates determined by conventional hybridizations. Each type of analysis, real-time PCR and conventional hybridization, were performed many times and, overall, we have greater confidence in the results from conventional hybridizations. By this method, the soybean cv Harosoy 63 was estimated to posses 27 ± 5 copies of *Hps *per haploid genome.

Variation in *Hps *copy number among different soybean lines could also be detected by comparative genomic hybridization (CGH) to cDNA microarrays. Although substantial differences in *Hps *copy number were detected by CGH, quantifying the number of *Hps *copies in a particular genome was not possible since hybridization intensities were not calibrated. Nonetheless, we have shown that CGH may be used to search for copy number polymorphisms in plant genomes. It is a potentially powerful application of microarrays that may be under-appreciated. For example, genomic DNA from plant lines that differ in a particular trait of interest could be screened using microarrays to identify genes that show differences in copy number. These genes could be tested as candidates for the trait of interest.

From our analysis of *Hps *gene structure, at least three pieces of evidence suggest that most of the *Hps *copies share a high degree of sequence identity. First, the hybridization patterns produced upon digestion of genomic DNA with a variety of enzymes indicate that restriction enzyme sites have been conserved in most of the gene copies. Secondly, analysis of *Hps *genomic clones indicates that independent clones with nearly identical sequences correspond to separate copies of *Hps *genes. Finally, expressed sequence tags encoding *Hps *transcripts do not show a high degree of sequence polymorphism [14,15]. Thus, it appears that most copies of the *Hps *gene have not diverged in sequence. This indicates that duplication and expansion of this gene cluster has been a recent event, or that sequence identity is maintained by frequent recombination events occurring within the cluster. Naturally, it would be desirable to clone a contiguous region of genomic DNA encompassing the entire tandem array of *Hps *genes. We attempted to do this by screening bacterial artificial chromosome (BAC) libraries but were unsuccessful. It is known that tandem arrays may be intractable to cloning and propagation [16], perhaps explaining this result.

In the cross between soybean lines OX281 and Mukden, *Hps *copy number polymorphisms cosegregated with seed lustre phenotype *B *and associated genetic markers. This result was expected because past studies have shown that *B *cosegregates with the presence of HPS protein on the seed surface, and with a DNA marker derived from the *Hps *cDNA sequence [2]. The multiple copies of *Hps *that are present in OX281 segregate in a Mendelian fashion, indicating that they occur at a single genetic locus and are not distributed throughout the genome. The analysis and assembly of *Hps *genomic clones substantiates the inheritance results, since the clones could be aligned to produce a reiterated array of *Hps *genes. All of the evidence therefore points to a tandem array of *Hps *genes occurring in a structural configuration arising from gene amplification.

Gene amplification occurs when multiple identical copies of a DNA sequence are duplicated within the genome. It may be an adaptive mechanism that results from selective pressure on the genome, as illustrated by drug, insecticide, or herbicide resistance observed in cell lines or in populations [17-19]. Amplification typically leads to a tandem array of reiterated units, such as that observed for genes encoding rRNA, snRNA, and histones [17]. Unlike genes that undergo duplication and divergence [20], individual units within a tandem array are under constraint and maintain a high-degree of sequence identity. Structural genes occurring in tandem arrays that are stable over generations, such as rDNAs, are considered a mechanism to accommodate cellular demand for large amounts of identical gene product. Genetic components embedded within tandem arrays that act to stabilize or promote gene amplification have been proposed, such as AT-rich tracts, autonomously replicating sequence (ARS) elements, and matrix attachment regions (MAR) [17]. These cis-acting elements have even been used to modulate gene copy number and expression levels of heterologous genes in transformed cells [21].

Thus, the features of the *Hps *locus appear to be consistent with characteristics associated with other amplified genes, from plants and animals. Plant genomes are known to have many large gene families and duplicated genes occurring in tandem arrays are also fairly common [22]. One of the largest tandem arrays characterized in plants corresponds to a gene cluster of 22 copies encoding alpha zeins in *Zea mays *[23], but there are few other examples of extensive arrays of nearly identical structural genes at one locus. The *Hps *locus is also exceptional because of the allelic variation in copy number of this gene cluster among different soybean cultivars and lines. Although it is not clear whether all copies of *Hps *are functionally expressed and transcribed, previous work has shown that transcripts encoding *Hps *are far more abundant in the endocarp of soybean lines with many genomic copies of *Hps *than in lines with few copies [5].

The results from this study together with past work [2,5] can be integrated into a model, whereby *Hps *genomic copy number operates as a genetic rheostat to control transcriptional and translational flux and the resulting quantity of HPS protein synthesized by the endocarp. Variation in HPS protein levels expressed in the endocarp could then account for the variable pattern of attachment of this tissue to the seed surface, and the resulting seed lustre phenotypes. Alternative explanations cannot be excluded, but the evidence so far tends to favour this gene amplification-based hypothesis. What kind of selection pressure could cause this to occur? The size, shape, colour, and general appearance of the seed are traits that are under intense selective pressure for crop plants, especially so for legumes. Even today certain markets may favour dull- or shiny-seeded soybeans for particular uses, so it is not unreasonable to suppose that selection for various lustre phenotypes has accompanied the development and expansion of this crop since its domestication some 3,000 years ago [24].

## Conclusion

This study demonstrates that copy number polymorphisms of the *Hps *gene are common in soybean cultivars and lines. In some cultivars, in excess of 27 ± 5 copies of *Hps *occur in a tandem array at a single locus. From these results, together with past studies on the occurrence and inheritance of the HPS protein, we developed a model to account for variation in seed lustre controlled by the *B *locus. The model proposes that *Hps *copy number changes provide a mechanism to modulate HPS protein levels expressed in the pod endocarp. Variable HPS expression in the endocarp likewise generates variation in the quantity and pattern of attachment of the endocarp to the seed surface, thereby affecting the seed lustre. Experiments in the future can be designed to test this model, and to investigate additional genetic loci controlling seed lustre that are independent of *B *and *Hps*.

## Methods

### Plant materials

Seeds of soybean (*Glycine max*) and *Lotus japonicus *were from collections at Agriculture and Agri-Food Canada, and were provided by Dr. Vaino Poysa and Dr. Krzysztof Szczyglowski, respectively. Seeds of *Glycine soja *and *Glycine tabacina *were from the USDA Soybean Germplasm Collection. Seeds of *Glycine canescens*, *Glycine curvata*, and *Glycine tomentella *were from the Australian National Herbarium. Plants were grown in field plots outdoors or in glass enclosed greenhouses. The cross of soybean line OX281 to cv Mukden and the generation of F_3 _families has been described [2]. Seed lustre was determined by visual inspection.

### Extraction and analysis of DNA samples

Soybean genomic DNA was purified from frozen tissues using a modified CTAB (hexadecyltrimethyl ammonium bromide) method [25]. Restriction enzyme digestion, electrophoretic separation on agarose gels, and blotting to Nylon membranes followed standard protocols [26]. Agarose gels were stained with ethiduim bromide and examined prior to transfer to ensure equal DNA loading and digestion. To prepare probes, the *Hps *cDNA was excised by restriction enzyme digestion from a plasmid clone [5]. Other probes were prepared by polymerase chain reaction (PCR) using cloned genomic fragments from soybean as DNA template (primer sequences and PCR conditions are available upon request). The DNA probes were isolated by excision from agarose electrophoresis gels, purified, and labelled with ^32^P dCTP using a random primer labelling system (RediprimeII, Amersham Biosciences, Baie d'Urfé, Canada). Unincorporated ^32^P dCTP was removed by gel filtration (Microspin S-200, Amersham Biosciences, Baie d'Urfé, Canada). Probes were denatured by heating to 95°C for 10 min. Hybridization was carried out at 65°C for 16 h in 0.25 mM Na_2_HPO_4_, pH 7.2, 7% (w/v) SDS, 1% BSA, and 1 mm EDTA. Filters were then washed four times for 15 min each at 22°C in high stringency wash solution (20 mM Na_2_HPO_4_, pH 7.2, 1% [w/v] SDS, and 1 mM EDTA), followed by three 15-min washes in the same solution at 68°C.

### Microarray analysis

Microarray slides (soybean 18 K – A series) were purchased from Dr. Lila Vodkin, University of Illinois, Urbana, IL. The slides contain 18613 soybean cDNAs of low redundancy, sourced from a variety of tissues and organs [27]. A total of six slides were hybridized in three separate experiments, using independent samples of genomic DNA for each experiment. Slides were prehybridized for 45 min in 5× SSC, 0.1% SDS and 1% BSA at 42°C, followed by two washes in 0.1× SSC at 22°C. The slides were dipped in water and dried by centrifugation. Genomic DNA purified from soybean tissues was digested with *Dpn *II and precipitated with ethanol prior to labelling with Cy3- or Cy5-dCTP (Amersham Biosciences, Baie d'Urfé, Canada) using published methods [13]. Labelled DNA was purified by column chromatography, quantitated, and the amount of dye incorporation was determined [28]. For hybridization mixtures, proportional amounts of Cy3- and Cy5-labelled DNA were dried by vacuum centrifugation, then re-dissolved and combined in a solution containing 22 ng uL^-1 ^mouse COT-1 (Invitrogen, Rockville, MD), 4.5 ng uL^-1 ^polyA DNA, 1× hybridization buffer (Amersham Biosciences, Baie d'Urfé, Canada), and 50% formamide (v/v). The hybridization solution (44 uL total volume) was denatured for 2 min at 100 °C, cooled for 30 min at 22°C, then applied to the prehybridized microarray slide. A 24 × 60 mm cover slip was placed over the slide and hybridization was carried out for 45 h in darkness, at 42°C. Post hybridization washes were as follows: one wash of 2× SSC and 0.1% SDS at 42°C for 5 min; two washes of 0.1× SSC and 0.1% SDS at 42°C for 2 min; and two washes of 0.1× SSC at 22°C for 1 min. Slides were dipped in water and dried under a stream of nitrogen gas prior to laser scanning (BioRad ChipReader with VersArray ChipReader v3.0 software, BioRad Corporation, Hercules, CA). Spot intensities were quantified and corrected for background signals (Array Vision v6.0 software, St. Catherines, ON, Canada), then values imported into GeneSpring v7.2 (Silicon Genetics, Redwood City, CA) and normalized by per spot, per chip, intensity dependant LOWESS. A dye swap was included in each experiment, and the final normalized hybridization ratios were averaged from separate values calculated for each slide. Data was MIAMI validated and deposited to the Gene Expression Omnibus [15] series GSE3810; samples GSM87407-GSM87412.

### Isolation and sequencing of genomic clones encoding *Hps*

Methods for construction and screening of genomic DNA libraries of soybean cv Harosoy 63 have been described [29]. Additional genomic libraries were prepared for this study, using commercially available λ-phage vectors and packaging extracts (Stratagene, La Jolla, CA), and by following the manufacture's instructions. Positive clones were plaque purified and sub-cloned into a plasmid vector (pBluescript, Strategene, La Jolla, CA) for sequence analysis. Automated sequencing of DNA was accomplished using dye-labelled terminators and fragments separated in acrylamide gels (model 377, Applied Biosystems, Foster City, CA). Genomic clones were shotgun sequenced by random transposon insertion (GPS-1, New England Biolabs, Beverly, MA) to an average of 10-fold coverage, and gaps were closed by primer walking. Finished sequences were assembled and edited using a computer program (Lasergene, DNAStar, Inc., Madison, WI).

## Authors' contributions

MG conceived of the research and wrote the manuscript. KK and PM performed the experiments. All authors read and approved the final manuscript.

## Figures and Tables

**Table 1:** A summary genomic DNA hybridizations to cDNA microarrays.

Experiment	Slide ID	Cy3 labelled DNA	Cy5 labelled DNA	Normalized hybridization ratios (OX281:Mukden or Harosoy 63:Sooty)	
				
				All spots (mean ± stdev)	Gm-r1070-2255 (*Hps*)
I	148927	Mukden	OX281		
	148932	OX281	Mukden	1.101 ± 0.594	5.537
II	148943	Mukden	OX281		
	148944	OX281	Mukden	1.011 ± 0.300	4.208
III	148945	Harosoy 63	Sooty		
	148946	Sooty	Harosoy 63	1.055 ± 0.516	6.406

Genomic DNA samples were individually labelled with fluorescent dyes Cy3 and Cy5, mixed, and hybridized to an 18 K array of soybean cDNAs. For each experiment, normalized hybridization ratios are averaged from separate values calculated for each slide, as described in Methods. The spot corresponding to cDNA clone Gm-r1070-2255 encodes an *Hps *transcript. Complete data set is accessible in Gene Expression Omnibus (GEO), series GSE3810.

**Table 2:** A summary of *Hps *genomic clones.

Clone name	GenBank accession number	Restriction enzyme fragment	Length (bp)	Probe used to isolate clone^1^	Description
*HPS2.1*	DQ208938	*Eco*RI	7663	Probe I	Related gene, contains ORF similar to HPS
*HPS1.5*	DQ208939	*Eco*RI	8042	Probe I	Contains complete ORF of HPS
*HPS1.6*	DQ208940	*Eco*RI	8086	Probe I	Contains complete ORF of HPS
*HPS1.11*	DQ208941	*Bam*HI	4733	Probe II	Contains partial ORF of HPS
*HPS1.13*	DQ208942	*Bgl*II	6049	Probe II	HPS intergenic or flanking region
*HPS1.15*	DQ208943	*Bgl*II	6193	Probe II	HPS intergenic or flanking region

The restriction enzyme fragments were isolated from genomic libraries of soybean cv Harosoy 63.

^1^The DNA probes correspond to those shown in Figure 6.

## References

[B1] Woodworth CM (1933). Genetics of the soybean. J Am Soc Agron.

[B2] Gijzen M, Weng C, Kuflu K, Woodrow L, Yu K, Poysa K (2003). Soybean seed lustre phenotype and surface protein co-segregate and map to linkage group E. Genome.

[B3] Wolf WJ, Baker FL, Bernard RL (1981). Soybean seed coat structural features: pits, deposits and cracks. Scannning Electron Microscopy.

[B4] Ma F, Cholewa E, Mohamed T, Peterson CA, Gijzen M (2004). Cracks in the palisade cuticle of soybean seed coats correlate with their permeability to water. Ann Bot.

[B5] Gijzen M, Miller SS, Kuflu K, Buzzell RI, Miki BLA (1999). Hydrophobic protein synthesized in the pod endocarp adheres to the seed surface. Plant Physiol.

[B6] Goudong Z, Jinling W, Qingxi M (1987). Inheritance of bloom on the seed coat in soybean. Soybean Genetics Newsl.

[B7] Chen Z, Shoemaker RC (1998). Four genes affecting seed traits in soybeans map to linkage group F. J Hered.

[B8] González R, Varela J, Carreira J, Polo F (1995). Soybean hydrophobic protein and soybean hull allergy. Lancet.

[B9] Antó JM, Sunyer J, Rodriguez-Roisin R, Suarez-Cervera M, Vazquez L (1989). Community outbreaks of asthma associated with inhalation of soybean dust. N Engl J Med.

[B10] González R, Duffort O, Calabozo B, Barber D, Carreira J, Polo F (2000). Monoclonal antibody-based method to quantify Gly m 1. Its application to assess environmental exposure to soybean dust. Allergy.

[B11] Gijzen M, González R, Barber D, Polo F (2003). Levels of airborne Gly m 1 in regions of soybean cultivation. J Allergy Clin Immunol.

[B12] Arumuganathan K, Earle ED (1991). Nuclear DNA content of some important plant species. Plant Mol Biol Rep.

[B13] Pollack JR, Perou CM, Alizadeh AA, Alizadeh AA, Eisen MB, Pergamenschikov A, Williams CF, Jeffrey SS, Botstein D, Brown PO (1999). Genome-wide analysis of DNA copy-number changes using cDNA microarrays. Nat Genetics.

[B14] The Institute for Genomic Research. http://www.tigr.org.

[B15] National Center for Biotechnology Information. http://www.ncbi.nih.gov.

[B16] Song J, Dong F, Lilly JW, Stupar RM, Jiang J (2001). Instability of bacterial artificial chromosome (BAC) clones containing tandemly repeated DNA sequences. Genome.

[B17] Graham GJ (1995). Tandem genes and clustered genes. J Theor Biol.

[B18] Paton MG, Karunaratne SH, Giakoumaki E, Roberts N, Hemingway J (2000). Quantitative analysis of gene amplification in insecticide-resistant Culex mosquitoes. Biochem J.

[B19] Widholm JM, Chinnala AR, Ryu JH, Song HS, Eggett T, Brotherton JE (2001). Glyphosate selection of gene amplification in suspension cultures of 3 plant species. Physiol Plant.

[B20] Bhattacharyya MK, Narayanan NN, Gao H, Santra DK, Salimath SS, Kasuga T, Liu Y, Espinosa B, Ellison L, Marek L, Shoemaker R, Gijzen M, Buzzell RI (2005). Identification of a large cluster of coiled coil-nucleotide binding site-leucine rich repeat-type genes from the *Rps1 *region containing *Phytophthora *resistance genes in soybean. Theor Appl Genet.

[B21] Borisjuk N, Borisjuk L, Komarnytsky S, Timeva S, Hemleben V, Gleba Y, Raskin I (2000). Tobacco ribosomal DNA spacer element stimulates amplification and expression of heterologous genes. Nat Biotechnol.

[B22] Zhang L, Gaut BS (2003). Does recombination shape the distribution and evolution of tandemly arrayed genes (TAGs) in the *Arabidopsis thaliana *genome?. Genome Res.

[B23] Song R, Llaca V, Linton E, Messing J (2001). Sequence, regulation, and evolution of the maize 22-kD alpha zein gene family. Genome Res.

[B24] Hymowitz T (1970). On the domestication of the soybean. Econ Bot.

[B25] Murray MG, Thompson WF (1980). Rapid isolation of high-molecular weight plant DNA. Nucl Acids Res.

[B26] Sambrook J, Fitsch EF, Maniatis T (1989). Molecular Cloning: A Laboratory Manual.

[B27] Vodkin LO, Khanna A, Shealy R, Clough SJ, Gonzalez DO, Philip R, Zabala G, Thibaud-Nissen F, Sidarous M, Stromvik MV, Shoop E, Schmidt C, Retzel E, Erpelding J, Shoemaker RC, Rodriguez-Huete AM, Polacco JC, Coryell V, Keim P, Gong G, Liu L, Pardinas J, Schweitzer P (2004). Microarrays for global expression constructed with a low redundancy set of 27,500 sequenced cDNAs representing an array of developmental stages and physiological conditions of the soybean plant. BMC Genomics.

[B28] Moy P, Qutob D, Chapman BP, Atkinson I, Gijzen M (2004). Patterns of gene expression upon infection of soybean plants by *Phytophthora sojae*. Mol Plant Microbe Interact.

[B29] Gijzen M (1997). A deletion mutation at the *ep *locus causes low seed coat peroxidase activity in soybean. Plant J.

